# Stretch Activates Human Myometrium via ERK, Caldesmon and Focal Adhesion Signaling

**DOI:** 10.1371/journal.pone.0007489

**Published:** 2009-10-16

**Authors:** Yunping Li, Maya Reznichenko, Rachel M. Tribe, Philip E. Hess, Michael Taggart, HakRim Kim, Jon P. DeGnore, Samudra Gangopadhyay, Kathleen G. Morgan

**Affiliations:** 1 Department of Anesthesia, Critical Care and Pain Medicine, Beth Israel Deaconess Medical Center, Harvard Medical School, Boston, Massachusetts, United States of America; 2 Boston Biomedical Research Institute, Watertown, Massachusetts, United States of America; 3 Division of Reproduction and Endocrinology, King's College London, London, United Kingdom; 4 Institute of Cellular Medicine, Newcastle University, Newcastle upon Tyne, United Kingdom; 5 Health Sciences Department, Boston University, Boston, Massachusetts, United States of America; 6 Department of Physiology, Tufts University School of Medicine, Boston, Massachusetts, United States of America; Sun Yat-Sen University, China

## Abstract

An incomplete understanding of the molecular mechanisms responsible for myometrial activation from the quiescent pregnant state to the active contractile state during labor has hindered the development of effective therapies for preterm labor. Myometrial stretch has been implicated clinically in the initiation of labor and the etiology of preterm labor, but the molecular mechanisms involved in the human have not been determined. We investigated the mechanisms by which gestation-dependent stretch contributes to myometrial activation, by using human uterine samples from gynecologic hysterectomies and Cesarean sections. Here we demonstrate that the Ca requirement for activation of the contractile filaments in human myometrium increases with caldesmon protein content during gestation and that an increase in caldesmon phosphorylation can reverse this inhibitory effect during labor. By using phosphotyrosine screening and mass spectrometry of stretched human myometrial samples, we identify 3 stretch-activated focal adhesion proteins, FAK, p130Cas, and alpha actinin. FAK-Y397, which signals integrin engagement, is constitutively phosphorylated in term human myometrium whereas FAK-Y925, which signals downstream ERK activation, is phosphorylated during stretch. We have recently identified smooth muscle Archvillin (SmAV) as an ERK regulator. A newly produced SmAV-specific antibody demonstrates gestation-specific increases in SmAV protein levels and stretch-specific increases in SmAV association with focal adhesion proteins. Thus, whereas increases in caldesmon levels suppress human myometrium contractility during pregnancy, stretch-dependent focal adhesion signaling, facilitated by the ERK activator SmAV, can contribute to myometrial activation. These results suggest that focal adhesion proteins may present new targets for drug discovery programs aimed at regulation of uterine contractility.

## Introduction

In late pregnancy increasing fetal growth significantly increases uterine wall tension. Compared to the nonpregnant uterus, human uterine weight increases from 70 grams to about 1100 grams at term pregnancy. Its total volume averages about 5000 ml, an expansion in size of approximately 250 fold [1]. No other smooth muscle organ in the human is able to stretch as much as the uterus. Myometrial stretch has been implicated, clinically, in the activation of the myometrium for labor, but the mechanisms involved are unclear. For example, it is known that multiple gestation pregnancies and polyhydramnios, conditions associated with increased tension/stretch on the uterine wall, cause an increased incidence of premature labor. Understanding the molecular basis of uterine contraction will aid the better control and manipulation of uterine contractile function in preterm and dysfunctional labor.

Focal adhesion complexes (also called dense plaques in smooth muscle) connect the intracellular cytoskeleton to the extracellular matrix and are recognized sites of mechanotransduction [2]. Previous studies [3], [4] in rodent myometrium have demonstrated that focal adhesion signaling is activated at late pregnancy. The authors suggested that neuronal and hormonal pathways alone may not be sufficient to bring about myometrial activation for labor and that a synergy of neuronal-hormonal pathways and mechanotransduction pathways could play an important role in parturition. Current knowledge of the roles of mechanical stretch in uterine regulation is based on data from animal models, and little information is available as to how this system works in human myometrium. Furthermore, the identity of signaling molecules involved in mechanotransduction pathways in human myometrium is little studied [5].

We have recently reported in the timed pregnant rat model that mechanical stretch of pregnant uterine smooth muscle activates ERK via focal adhesion signaling. In the rat, we have shown that in addition to classical GPCR-mediated pathways, this ERK pathway, in a cause-and-effect manner, facilitates myometrial contraction, and plays a distinct role in the switch from the quiescent phase of pregnancy to a more contractile phenotype at the end of pregnancy [6], [7].

Smooth muscle archvillin (SmAV) is a regulator of ERK pathways newly identified by our group [8], [9]. It is a member of the supervillin family that is preferentially expressed in smooth muscle and was first identified as an interactor of the smooth muscle differentiation marker, h-1 calponin in a 2-hybrid assay. Its function in myometrium has not been previously studied.

In the present study, we tested the hypothesis that the stretch-mediated activation of focal adhesion signaling molecules occurs during human pregnancies and describe, for the first time, an up-regulation during gestation and association with focal adhesion complexes of the ERK regulator, SmAV, in human myometrium.

## Materials and Methods

### Human Myometrial Tissue Collection

Ethics Statement: The consent forms for human myometrial tissue collection were approved by the Committee on Clinical Investigations at Beth Israel Deaconess Medical Center and Central Manchester Healthcare Trust LREC (UK). The human pregnant myometrial samples were taken from the upper edges of the lower segment uterine incision during Cesarean sections (n = 44) or *ex-utero* intrapartum treatment (EXIT) procedures (n = 3). The nonpregnant human myometrial samples (n = 8) were collected from pre-menopausal patients undergoing hysterectomy to remove the uterus for benign gynecological conditions and samples were obtained at the uterine lower segment. Exclusions for sample collection were patients with a history of hypertension or pregnancy-induced hypertension who are on antihypertensive medication, those with preterm labor who are currently on medication treatment, or patients undergoing emergent cesarean section from whom unable to obtain a formal consent.

### Rat Myometrial Tissue Collection

All procedures were approved by the Boston Biomedical Research Institute Animal Care and Use Committee and complied with he American Physiological Society “Guiding Principles for Research Involving Animals and Human Beings”. Sprague-Dawley nonpregnant and timed-pregnant rats (Taconic, Germantown, NY) were euthanized by carbon dioxide inhalation. Delivery was observed to occur on the 22nd or 23rd gestational day. For the collection of in-labor uterine smooth muscle samples, the rat was closely observed and the delivery of the first pup was used as the indication of labor. Excised uteri were immersed immediately into oxygenated Krebs solution at room temperature. The details of tissue handling and protein extraction have been published previously [6], [10].

### Tissue Preparation and Force Recording

The whole-thickness human uterine smooth muscle strips were microdissected to a size of approximately 10×3×3 mm, and oriented parallel to the longitudinal axis of muscle bundles. Four myometrial strips were dissected from each sample for in vitro stretch experiments. The satisfactory condition of each uterine smooth muscle strip was confirmed before the experiment by the presence of active, spontaneous contractions with or without 51 mM KCl treatment. Isometric force was recorded at 37°C as previously described [10]. For in vitro stretch experiments, the strips were stretched to 2× slack length. Tension was applied gradually over 20–30 seconds and maintained for the duration of experiment. The contractile activity was digitalized with MacLab/8e, Chart v3.5.4 (AD Instrument, Castle Hill, Australia).

All the samples used for in vitro stretch experiments were term, not in labor human pregnant myometrium. The justification for stretch of 2× slack length is based on the optimal length of term pregnant uterine smooth muscle strips in the rat model [7], [10] and the pilot stretch experiments on human myometrium. Also, this optimal length is same as in vivo length as measured when the uterine wall is stretched by the fetus in the rat model. Clinically, it is impossible to measure the in vivo length of the uterine smooth muscle that will be sampled later. In general, in vitro stretch to 2× slack length generates force in the range of 10–15 grams. This is about a 20-fold increase in force compared to baseline tension of term, not-in-labor uterine strips in the organ bath. The area under the curve (AUC) was obtained by integrating the force signal (in gram) over indicated time period (in second).

### Immunoblotting

Strips were quick-frozen at different time points after a mechanical stretch in a dry ice/trichloroacetic acid/acetone slurry containing 10 mM DTT. The frozen samples were homogenized as previously described [6]. Protein concentrations were quantified by modified Lowry protein assay (DC Protein Assay Kit, Bio-Rad). Protein-matched samples were boiled, separated by SDS-PAGE, transferred to a PVDF membrane and analyzed by immunoblotting with appropriate antibodies.

For caldesmon (CaD) and rat SmAV protein content quantification, the blots were visualized with a SuperSignal West Pico peroxide solution (Pierce, Rockford, IL). The images were detected with a chemiluminescence screen and quantified with a Bio-Rad Phospho Imager and Multi-Analyst software. In rest experiments, the intensity of Western blot signals was quantitated by using an Odyssey Infrared Imaging System (LI-COR Bioscience, Lincoln, Nebraska). To minimize the variation of the signal intensity between different Western blotting experiments, we developed blots to similar intensities or used a reference sample in each blot for normalization if multiple samples were used from different pregnant women.

### Calcium sensitivity in alpha-toxin permeabilized human myometrial strips

The method was identical to the method previously published [10]. The force of contractions generated at 10^−7^ M free calcium concentration were recorded and expressed as a percentage of maximal contraction at pCa 5. This measurement was used as an index of calcium sensitivity.

### Immunoprecipitation

The myometrial smooth muscle strips were frozen in a dry ice/acetone slurry containing DTT. The tissue was pulverized with a mortar and pestle cooled with liquid nitrogen. The tissue powder was dissolved in a buffer containing 50 mM Tris (pH 7.4), 5 mM EGTA, 140 mM NaCl, 1% NP40, 1% Na deoxycholate, 20KIU aprotinin, 5 µM leupeptin, 5 µM pepstatin A, 2 mM Na_3_VO_4_, and 1 mM NaF and centrifuged at 12,000 rpm at 4°C for 15 min. The supernatant was precleared with protein A agarose and then incubated with anti-phospho tyrosine or anti-ERK conjugated agarose beads overnight at 4°C with gentle rotation. The immune complex beads were washed, resuspended in a sample buffer (25 mM Tris (pH 6.8), 4% SDS, 20% glycerol, 5% β-mercaptoethanol, 1 mM EDTA and 0.001% bromophenol blue), and boiled at 100°C for 5 min. The proteins of interest were detected by western blotting with specific antibodies.

### Materials

An anti-SmAV antibody was produced from a bacterially expressed, His-tagged, fragment containing the first 250 N-terminal residues of ferret SmAV. The purified peptide was injected into rabbits and polyclonal antiserum was developed by Capralogics, Hardwick, MA. The antibody was affinity purified from the serum (AminoLink Kit, Pierce Biotechnology) and the purified antibody recognizes the antigen and also recognizes a single protein band of appropriate molecular weight and no other bands in aorta tissue homogenates. This antibody was used for immunoblot and imaging studies.

The CaD polyclonal antibody (1∶30,000) was raised against full-length human myometrial CaD and was a gift from Dr. K. Mabuchi (Boston Biomedical Research Institute). The phospho-CaD antibody (1∶500 Upstate, Lake Placid, NY) was produced against a phosphopeptide containing the CaD sequencing surrounding the Ser^789^ ERK phosphorylation site. The p44/42 MAP kinase1/2 (1∶1000), phospho p44/42 MAP1/2 kinase antibody (1∶1500), phospho-FAK (Y925, 1∶500) and phospho-p130Cas (1∶500) were purchased from Cell Signaling Technology (Beverly, MA). Phospho-tyrosine monoclonal antibody, clone 4G10 (1∶500–1500), anti-phospho-tyrosine and anti-MAP kinase ERK1/2 agarose-conjugated beads were the products of Upstate. P130Cas and Phospho-FAK (Y397, 1∶1000) antibodies were purchased from BD Transduction Laboratories (San Diego, CA). FAK (1∶300) was a polyclonal antibody from Santa Cruz Biotechnology Inc. (Santa Cruz, CA). Monoclonal alpha-actinin (1∶1000) antibody was purchased from Sigma.

### Mass Spectrometry

The protein band of interest was excised from one-dimensional, Coomassie blue stained PAGE gels. Mass spectrometry for protein identification was performed by the Tufts University Core Facility (Boston, Massachusetts). Excised bands were subjected to in-gel reduction, alkylation, and enzymatic digestion with trypsin (Roche Applied Science, Indianapolis, IN). LC/MS/MS analysis was performed on the in-gel digest extracts using an Agilent (Santa Clara, CA) 1100 binary pump directly coupled to a mass spectrometer. Nanobore electrospray columns were constructed from 360 µm o.d., 75 µm i.d. fused silica capillary with the column tip tapered to a 15 µm opening (New Objective, Woburn, MA) and were packed with 200 Å 5 µm C18 beads (Michrom BioResources. Auburn, CA.) to a length of 10 cm. The flow through the column was split pre-column to achieve a flow rate of 300 nL/min. The mobile phase used for gradient elution consisted of (A) 0.3% acetic acid 99.7% water and (B) 0.3% acetic acid 99.7% Acetonitrile. Tandem mass spectra (LC/MS/MS) were acquired on a Thermo LTQ ion trap mass spectrometer (Thermo Corp., San Jose, CA). The MS/MS spectra were searched against the NCBI non-redundant protein sequence database using the SEQUEST computer algorithm [11] to produce a list of proteins identified in each sample. Confident protein identification was determined by the Proteomics Browser software (Thermo Corp., San Jose, CA) and was defined as at least 4 unique peptides matching for a given protein.

### Immunohistochemistry

The whole-thickness, term, not-in-labor, human uterine smooth muscle strips were microdissected and stretched to 2 fold slack length for 7 minutes. Stretched muscles were fixed by immersion in 4% paraformaldehyde for 2 hours at 4°C and embedded with optimal cutting temperature compound (OCT, Tissue-Tek). Frozen samples were sliced 10 micrometers thick and stained with rabbit anti-SmAV and mouse anti-vinculin (Sigma). Alexa 568-conjugated goat anti-rabbit (red) and Alexa 488 goat anti-mouse (green) secondary antibodies (Molecular Probe Inc.) were used for staining. DAPI was used as a nuclei stain. Images of immunostained myometrium were taken with a Nikon Eclipse TE 2000-E inverted microscope equipped with a Nikon Plan Apochromat 60XA (NA 1.4) oil immersion objective. Images were recorded by a high-resolution fluorescence CCD camera (CoolSNAP™ HQ^2^, Photometrics®) with NIS-Elements Advanced Research (Nikon) software. In all cases, it was confirmed that there was no detectable background fluorescence by staining cells in the absence of a primary antibody. In addition, for all colabeling experiments, it was confirmed that there was no detectable cross talk between fluorescent labels by exchanging excitation/emission filters on single labeled samples. Resolution was optimized by deconvolution microscopy software. The algorithm used image information from different Z-slices (Richardson-Lucy algorithm, constrained iterative-maximum likelihood estimation algorithm).

### Statistics

Data were expressed as mean± SE. Significance of difference between two individual sets of means was taken at p<0.05 by an unpaired Student's *t* test unless indicated otherwise. In stretch experiments, a one-way ANOVA test was used for the time course study. The probability values of p<0.05 was considered significant.

## Results

### Human myometrial CaD protein content and the [Ca^2+^] requirement for contractile activation increase during pregnancy

**Figure 1:**
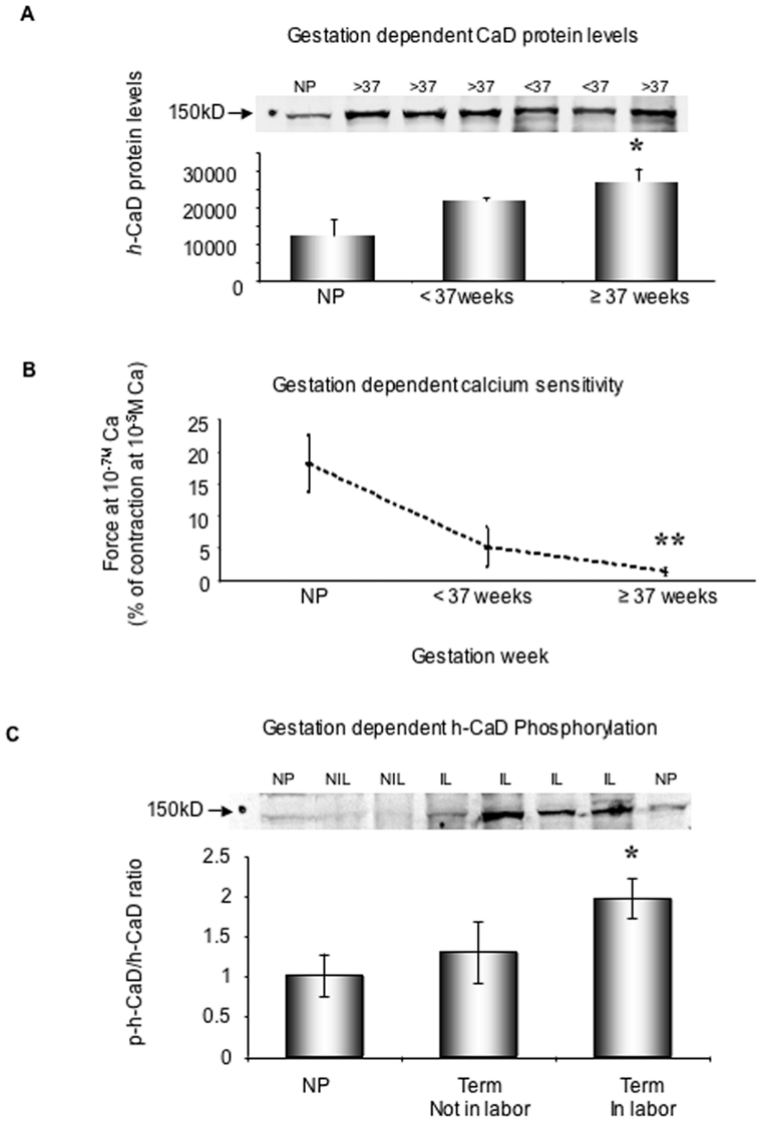
Gestation-dependent changes in CaD content, calcium sensitivity and CaD phosphorylation. A. *h-*CaD expression increases during pregnancy. n = 3–7 samples in each group. * p<0.05 compared to NP. NP: nonpregnant. A single representative blot is illustrated on the top. To minimize the variation of the signal intensity between different Western blotting experiments, a reference sample was used in each blot for normalization since multiple samples were used from different pregnant women. B. Myometrial calcium sensitivity decreases with gestation. Human nonpregnant and pregnant not in labor uterine strips were permeabilized with alpha-toxin. Contractile force at 10^−7^ M calcium is expressed as a percentage of contraction induced by 10^−5^ M calcium. n = 3–5 samples in each group ** p<0.001 compared to NP. C. Increased CaD phosphorylation at Ser^789^ occurs only with the onset of labor. Phosphorylated p-*h*-CaD is normalized to *h-*CaD protein level. A single representative blot is included on the top. NP: nonpregnant, NIL: term, not in labor, IL: term, in labor. The samples used in experiments for Fig 1A and Fig 1C are different. Figure 1C is an independent experiment. *p<0.05 vs. NP. n = 5–7 samples in each group.

h-Caldesmon (CaD) is a smooth muscle-specific actin binding protein that interferes with acto-myosin interactions [12]. In myometrial samples collected from pregnant women who underwent Cesarean sections or non-pregnant women who underwent gynecological procedures, we confirmed the earlier reports of Word et al [13] and Riley et al [14] that h-CaD protein expression levels increase significantly during pregnancy (26907±3535 in ≥37 weeks group vs. 13656±3752 in NP group)(Fig. 1A).

Since h-CaD is known to inhibit acto-myosin interactions in vitro [15], we tested the relative Ca sensitivity of activation of the contractile filaments of human myometrial preparations permeabilized with alpha toxin [10]. Shown in Figure 1B are the average contractile force responses of myometrial preparations from nonpregnant women, women pregnant for <37 weeks (not in labor) and those pregnant for ≥37 weeks (not in labor) to a [Ca^2+^] of 10^−7^ M, expressed as a percentage of the maximal force obtained at 10^−5^ M [Ca^2+^]. Calcium responsiveness of the contractile apparatus is significantly decreased in the late pregnancy samples compared to that of samples from non-pregnant women. Our data are consistent with the hypothesis that the observed increased inhibitory caldesmon protein content in human myometrium leads to a decreased contractile responsiveness to Ca during pregnancy. During a normal pregnancy the uterus is known to clinically display a “myometrial quiescence” before labor. This decreased Ca responsiveness of the contractile apparatus is one factor that could explain this quiescence during pregnancy.

### Gestation- dependent increases in h-CaD phosphorylation in human myometrium

In the timed pregnant rat model, we have shown that as labor begins, an ERK pathway is activated that phosphorylates CaD [10] and removes the inhibitory influences of CaD on actomyosin interactions [15] and, as a result contributes to the onset of labor [7]. Whether this ERK-mediated phosphorylation of h-CaD during labor occurs in the human has not been reported. We probed quickly frozen human myometrial samples from nonpregnant women, term pregnant women, but not in labor, and term pregnant women and in labor. We found (Fig. 1C) that h-CaD phosphorylation at an ERK specific site (Ser 789) increases significantly in human myometrial tissue from pregnant women in labor (1.97±0.24 vs. 1.01±0.27 in NP group) but not from those not in labor (1.3±0.39). These results are consistent with phosphorylation of CaD being a contributing factor for labor onset.

### Stretch of human myometrium causes increased ERK activation and h-CaD phosphorylation

**Figure 2:**
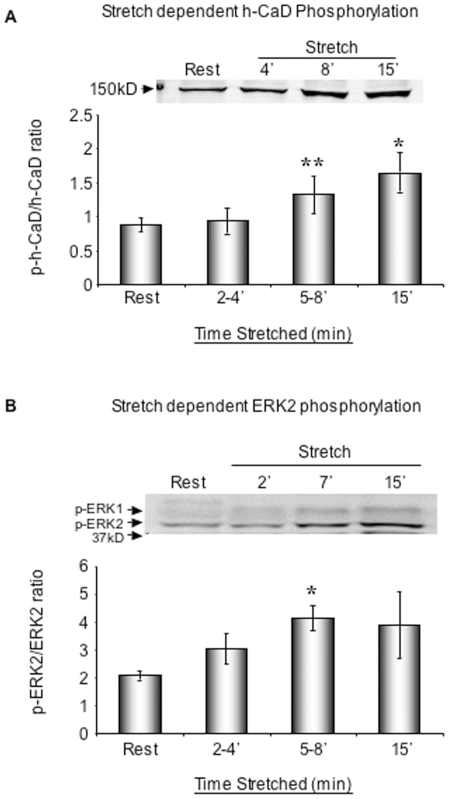
Stretch of human myometrium directly increases *h-*CaD and ERK phosphorylation. A. h-CaD phosphorylation increases in response to stretch. Phosphorylated p-*h*-CaD is normalized to *h-*CaD protein level. *p<0.05 and ** p = 0.01 compared to resting sample (by ANOVA). n = 4 samples in each group. B. Stretch increases ERK2 phosphorylation. p-ERK2 signals are normalized to the total ERK2 protein levels. *p<0.05 compared to resting samples (by ANOVA). n = 4–5 samples in each group.

The increased h-CaD phosphorylation seen at the end of term could be due to many factors including hormonal factors as well as the increased stretch of the myometrial wall. To test whether stretch itself directly regulates ERK-dependent h-CaD phosphorylation, we exposed human myometrial strips to in vitro stretch. Human myometrial strips (term, not-in-labor) were stretched to 2x slack length and quick frozen at indicated time points for the measurement of CaD phosphorylation by immunoblot. Strips at slack length were used as a control group. By 5–15 minutes after the onset of stretch, CaD phosphorylation, normalized for total CaD protein, significantly increased (Fig. 2A). Although the increase is modest, the change in CaD phosphorylation levels is statistically significant.

The phospho-CaD antibody used for the immunoblots is specific for CaD phosphorylation at a known [16] ERK phosphorylation site, Ser^789^, suggesting that ERK could be the kinase responsible for CaD phosphorylation and activation. To test this hypothesis, we monitored ERK activation in response to in vitro stretch by immunoblotting for phospho-ERK. As is shown in Figure 2B, in vitro stretch does increase ERK2 phosphorylation at Ser^789^ in a statistically significant manner. In contrast, phospho ERK1 levels did not change significantly in response to stretch (data not shown).

### Mechanical stretch in vitro increases myometrial contractility

**Figure 3:**
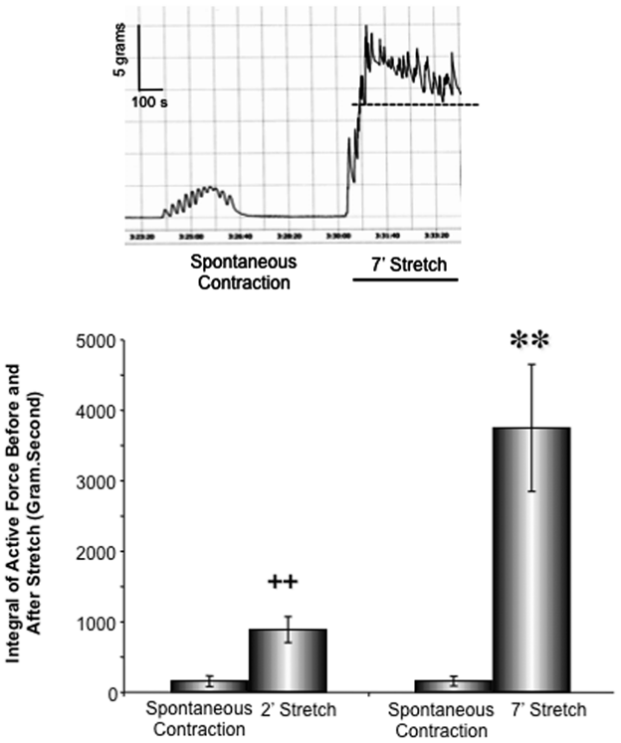
*In vitro* stretch increases contractility in pregnant myometrium. Uterine contractility was measured and quantified as the area under curve (AUC). AUC is the integral of the *active* force over a period time of 2 or 7 minutes before and after stretch. Dotted line indicates level of passive stretch. Data presented in the bar graph were collected from 8 term, not in labor, pregnant human myometrial samples and total 17 smooth muscle strips. ++ p<0.01 compared to the AUC of spontaneous contraction before 2 minute-stretch. ** p<0.01 compared to the AUC of spontaneous contraction before 7 minute-stretch. An insert was the representative myograph recording of contractile force from term pregnant human myometrium in response to *in vitro* stretch.

The above data demonstrate that the existence of stretch-sensitive elements in the pregnant myometrium that result in ERK and CaD activation. Our previous work in a rat model suggested that ERK activation and subsequent CaD activation lead to corresponding changes in contractility [6], [10]. This hypothesis was tested in current study with term, not in labor human myometrium. As shown in figure 3, *in vitro* stretch quickly switched the uterine smooth muscle from relatively quiescent status to an active contractile state, characterized by a significant increase in amplitude. The changes in contractility before and during stretch were quantified by measuring the area under the active tension curve above the level of passive tone (AUC). The uterine contractility increases in a highly significant manner in response to 2 and 7 minutes stretch *in vitro*.

### Stretch of human myometrium induces an increase in tyrosine phosphorylation and activation of focal adhesion signaling

The above data imply that gestational stretch due to the growing fetus, and, in vitro stretch imposed experimentally on the human myometrium, can induce ERK and CaD phosphorylation. Focal adhesions (FA) make direct contact with the extracellular matrix (ECM), providing a structural link between the ECM and actin cytoskeleton. FAs are mechano-sensors or transducers of “outside-in signaling” [17], [18], which is known, in non-muscle cells, to involve tyrosine phosphorylation of many proteins. In contrast, in differentiated, non-migrating smooth muscle cells, most signaling events regulating contractility involve Ser/Thr phosphorylation. Thus, as a first screen for adhesion plaque signaling events we probed human myometrial homogenates in the presence or absence of stretch with an anti-phosphotyrosine antibody.

**Figure 4:**
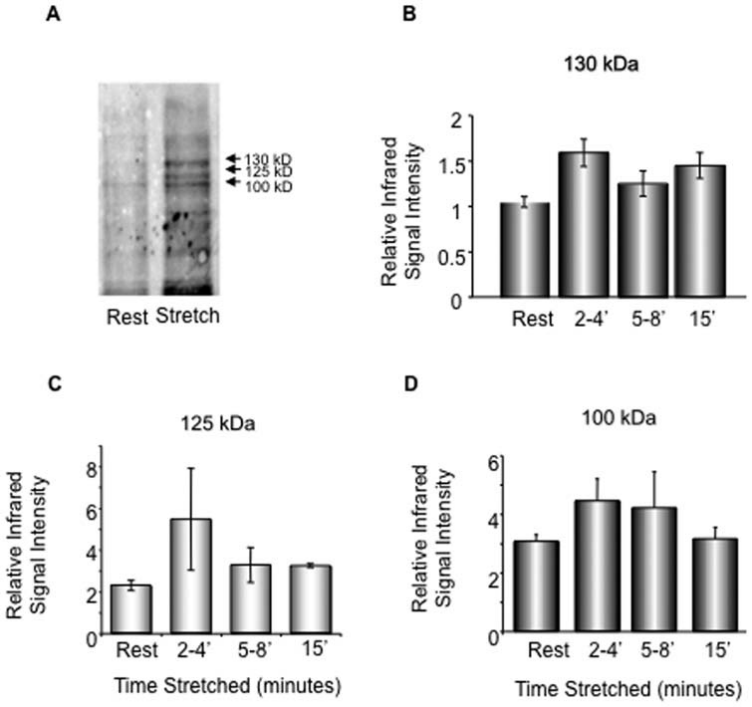
Stretch-induced tyrosine phosphorylation in human myometrium. A. A typical anti-phosphotyrosine immunoblot. Term pregnant uterine smooth muscle strips unstretched or stretched to 2x slack length for 2 min. The tissue homogenates were immunoblotted with an anti-tyrosine phosphorylation antibody. B–D. Term pregnant uterine smooth muscle strips were unstretched (rest) or stretched to 2x slack length at indicated time period. Average densitometry of phospho-tyrosine bands of 130 kD, 125 kD and 100 kD. n = 3–5 samples in each group.

As is seen in Fig. 4, there was a trend toward increases in the tyrosine phosphorylation of (approximately) 130 kDa, 125 kDa and 100 kDa bands with stretch, so we probed the human samples with protein-specific phospho-antibodies. Of note, in the pregnant rat model, only the 125 kDa band showed a pronounced increased tyrosine phosphorylation in this range of molecular weights [6]. The 125 kDa tyrosine phosphorylated protein in the timed pregnant rat model was identified as focal adhesion kinase, so we probed the human samples with protein-specific phospho-FAK antibodies.

**Figure 5:**
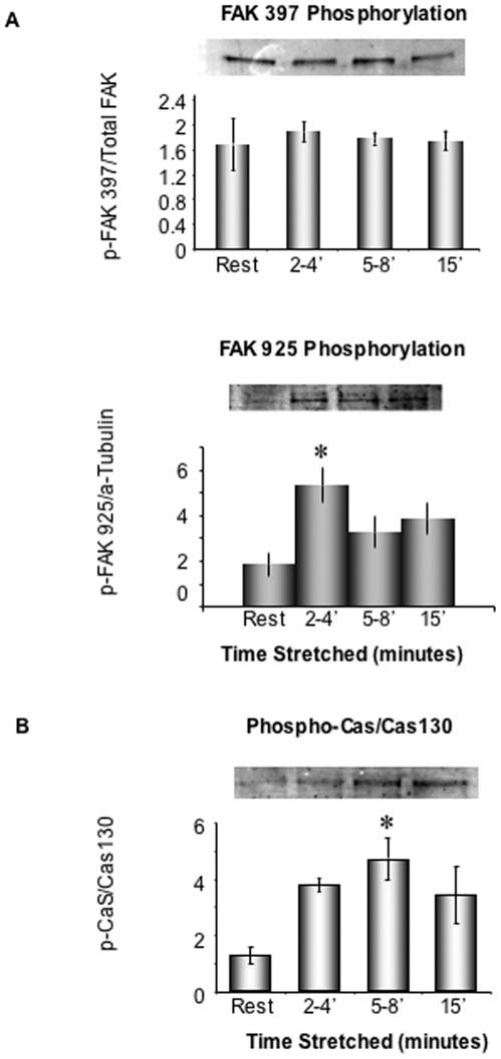
Identification of p125 as FAK and p130 as Cas. A. Stretch selectively activates FAK at tyrosine site 925, not site 397. Term pregnant uterine smooth muscle strips were unstretched or stretched to 2x slack length at indicated time period. The tissue homogenates were probed with FAK site-specific antibodies. Phospho-FAK signals are normalized to the total FAK protein or alpha-Tubulin. *p<0.05 compared to unstretched control samples (by ANOVA). n = 3–5 in each group. Typical blots were shown on the top. Both FAK total protein antibody and FAK-Y925 phospho antibody are rabbit polyclonal antibodies, thus, a-tubulin mouse monoclonal antibody was chosen for normalization of the FAK-Y925 signal. B. Stretch activates pCas130. Phospho-Cas signals are normalized to Cas protein levels and expressed as p-Cas/Cas ratios. *p<0.05 compared to unstretched control samples (by ANOVA). n = 3–5 samples in each group.

FAK phosphorylation at Y397 is thought to signal integrin engagement whereas phosphorylation at FAK-Y925 is thought to signal subsequent downstream signaling to an ERK pathway [19]. Interestingly, we could detect no change in FAK-Y397 phosphorylation during acute in vitro stretch of human term myometrial samples (Fig. 5A, top). It is possible that phosphorylation at this site was already activated in vivo, before the samples were obtained. However, acute stretch did cause a significant increase in FAK-Y925 phosphorylation (Fig. 5A, bottom) perhaps mimicking events that occur later *in vivo* during the switch to initiate labor.

p130 Cas is a well-known, stretch sensitive, adhesion plaque protein [20], [21], so we postulated that the 130 kDa molecular weight tyrosine phosphorylated band could be Cas and then probed with a Cas-specific phospho-antibody. The phospho-Cas signals were normalized to total Cas protein content and expressed as a ratio of p-Cas/Cas. As shown in figure 5B, Cas is significantly phosphorylated within 5–8 min in response to in vitro stretch in human myometrium.

Elevations of tyrosine phosphorylation of FAK and Cas appeared to be transient in the myometrial stretch experiments. This could be explained by the dynamic assembly and disassembly of focal adhesion complexes as a function of stretch. In contrast, the downstream of resulting serine/threonine phosphorylation of ERK/CaD may integrate upstream signals and be more sustained (Fig. 1C and Fig. 2).

Probing the 100 kDa band for candidates with similar molecular weights was unsuccessful so we turned to a mass spectrometry approach.

### Identification of proteins tyrosine phosphorylated in response to myometrial stretch by SDS/PAGE and Mass Spectrometry

**Figure 6:**
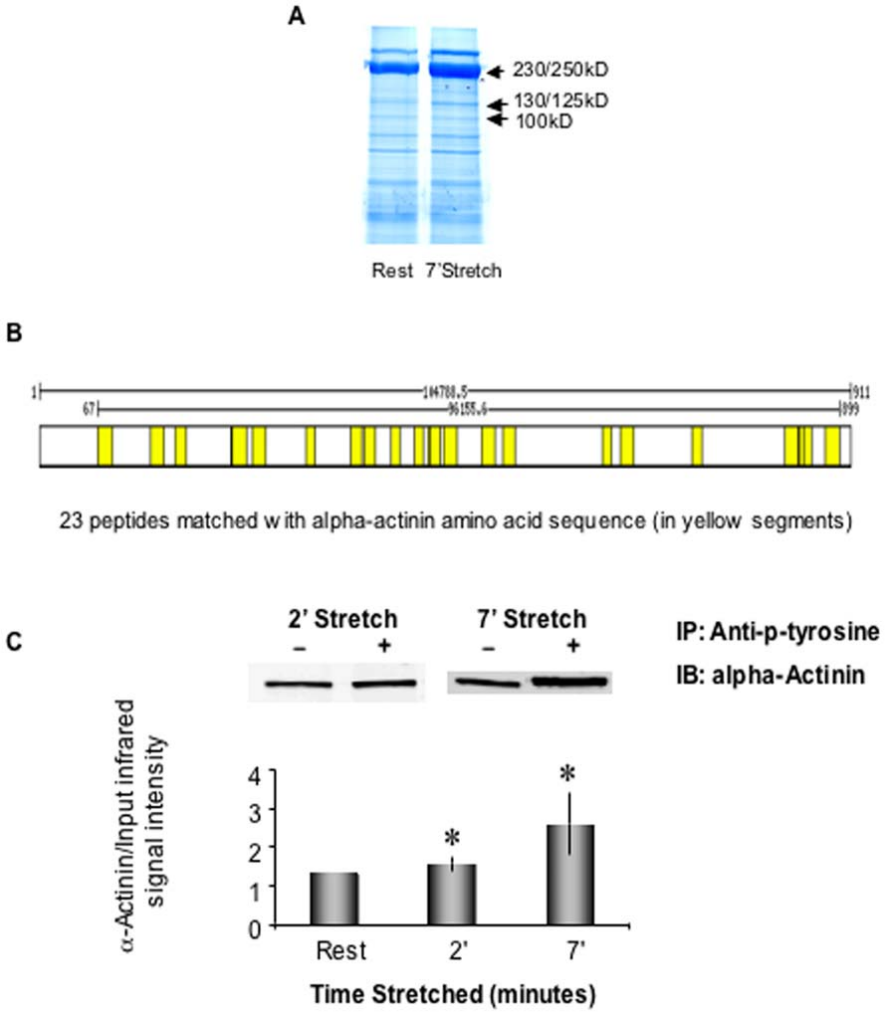
Identification of 100 KD protein tyrosine-phosphorylation in response to myometrial stretch. A. Stretch-induced tyrosine phosphorylation in human myometrium. Term pregnant uterine smooth muscle strips unstretched or stretched to 2x slack length for 7 min. Coomassie blue staining of anti-phosphotyrosine immunoprecipitates separated by 10% SDS-polyacrylamide gel electrophoresis (n = 2). B. Mass spectrometric identification of the 100 kD band. The protein band was excised from the gel. There is a 281/911 (31%) sequence coverage with 23 peptides matched to alpha 4 actinin, (in yellow) and 19% sequence coverage with 13 peptides matched to alpha 1 actinin (not shown). C. Alpha actinin is tyrosine phosphorylated by stretch in human myometrium. Homogenates of stretched and unstretched term pregnant human uterine smooth muscle were immunoprecipitated (IP) with an anti-phospho-tyrosine antibody and western immunoblotted (IB) with an alpha-actinin antibody. The graph shows the average densitometry from 5 independent experiments. The inserts are typical western blots of immunoprecipitates. *p<0.05 compared to unstretched control samples.

To further identify human myometrial proteins tyrosine phosphorylated in response to stretch, we immunoprecipitated proteins with an anti-phosphotyrosine antibody using protein-matched homogenates of unstretched and stretched myometrial strips (term pregnant, slack, or stretched 2x slack length for 2′ or 7 min). Fig. 6A shows a Coomassie blue stained SDS/PAGE gel of such immunoprecipitates. It is of note that many proteins in myometrial tissue from pregnant women are heavily tyrosine phosphorylated in the basal state. Bands at 250, 130 and 100 kDa that displayed a consistent increase in intensity (indicating either an increase in Tyr phosphorylation or a binding to such a protein in the immunoprecipitate) after stretch were subjected mass spectrometric analysis.

The 100 kDa protein band was found to contain both alpha actinin 1 (13 peptide matches) and alpha actinin 4 (23 peptide matches) isoforms. Fig. 6B illustrates the high degree of peptide coverage (31%) for the alpha actinin 4 isoform. The finding was confirmed by immunoblot of the 100 kDa anti-phosphotyrosine immunoprecipitates with an alpha actinin-specific antibody. As is shown in Fig. 6C, the amount of alpha actinin immunoprecipitated with an anti-phosphotyrosine antibody significantly increased with stretch, indicating either, that the protein itself is tyrosine-phosphorylated with stretch, or, that its association with other tyrosine-phosphorylated proteins increases with stretch. Alpha actinin is known to be present in adhesion plaques and dense bodies in smooth muscle where it links the extracellular matrix to the actin cytoskeleton and the contractile filaments. Alpha actinin is also known to be directly tyrosine phosphorylated in vitro, and the effect is reported to play a role in pressure-activated cell adhesion in colon cancer cells [22], [23]. Disruption of alpha-actinin-integrin interactions at focal adhesions renders osteoblasts susceptible to apoptosis. A truncated form of alpha-actinin (ROD-GFP) competitively displaces endogenous alpha-actinin from focal adhesions, disrupts focal adhesions and reduces tyrosine phosphorylation at focal adhesions. Thus alpha-actinin plays a role in regulating stabilization of focal adhesions [24] and its increased association with tyrosine phosphorylation could also be a contributing factor to the onset of labor.

In mass spectrometric analysis of the 250 kDa band from the phosphotyrosine immunoprecipitate strong matches to myosin heavy chain and to filamin A were found. Myosin is known to connect with adhesion plaques, and one isolated report has indicated that myosin heavy chains under some conditions can be tyrosine phosphorylated [25], but, we also cannot rule out the possibility of myosin contamination of the mass spectrometry samples given its abundance in smooth muscle. Filamin was also identified in this band and is known to be associated with adhesion plaques [26]. Filamin is also well known to be directly phosphorylated by Src kinases and associated with adhesion plaque turnover [27].

Analysis of the 130 kDa band that increased with stretch in the phosphotyrosine immunoprecipitates, gave a confident match for presence of caldesmon (CaD). Although tyrosine phosphorylation of l-CaD has been described, to the best of our knowledge, h-CaD has not been reported to be directly tyrosine phosphorylated. Furthermore, the 130 kDa isoform of h-CaD is known to be primarily located in contractile filaments. However, the detection of h-CaD in the phosphotyrosine immunoprecipitates may reflect the possible connection of the contractile filaments to the phosphotyrosine-containing adhesion plaques. We were unable to identify p130 Cas in these samples by mass spectrometry, probably because of the relatively low abundance of this signaling molecule compared to cytoskeletal proteins such as CaD, myosin, alpha actinin and filamin.

### Gestation-dependent increase in SmAV expression in both human and rat myometrial tissues

**Figure 7:**
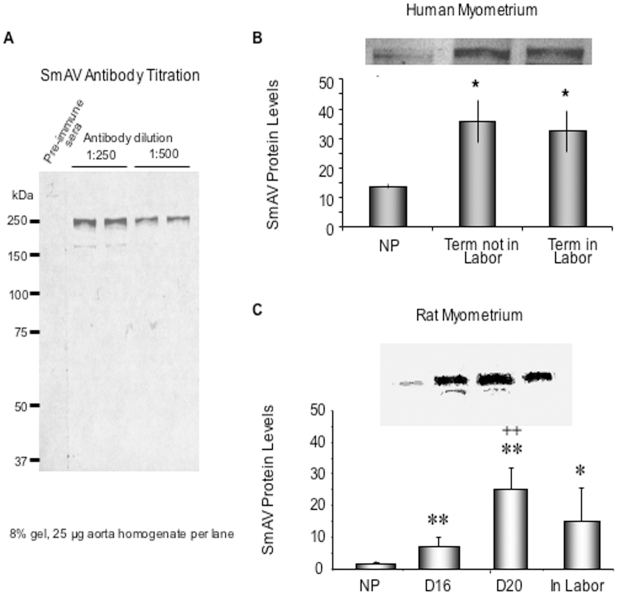
SmAV protein content increases with gestation in both the human and the rat. A. Specificity of the anti SmAV antibody. B. Gestation-dependent increase in SmAV expression in human myometrium. Densitometry analysis of smooth muscle Archvillin (SmAV) protein levels from immunoblots of human myometrium from nonpregnant (NP) and pregnant women. A typical blot is shown on the top. *p<0.05 vs. NP. n = 5–7 samples in each group. C. Gestation-dependent increase in SmAV expression in rat myometrium. A typical blot is shown on the top. *p<0.05 compared to NP, **p<0.01 compared to NP. ++ p<0.01 compared to D16. n = 4 in each group. NP, nonpregnant, D16 and D20, pregnant day 16 or day 20.

Smooth muscle archvillin (SmAV) is an ERK regulator newly identified in our laboratory [8], [9]. Its presence and function in myometrial smooth muscle have not been previously studied, but it has been shown to regulate both ERK activation and contractility in vascular smooth muscle [8]. Both SmAV and the founding member of the protein family, supervillin are associated with adhesion plaques [8], [28]. Thus, we postulated that SmAV might be involved in gestation- and stretch-sensitive changes in ERK activation in pregnancy and it might be the link between mechanical signal and activation of ERK/CaD pathway. We developed a polyclonal, affinity-purified antibody to a recombinant fragment of SmAV containing the first 250 N-terminal residues. Specificity of the antibody was tested against a whole tissue aorta homogenate and only a single band, at the expected molecular weight, was detected. In contrast, preimmune serum produced no bands in the same sample (Fig. 7A).

To test whether SmAV expression is gestation-dependent, myometrial strips from nonpregnant, pregnant, not in labor, and pregnant, in-labor women were quick-frozen. Densitometry of protein-matched samples were analyzed in anti-SmAV immunoblots. SmAV protein expression increases significantly during pregnancy in human myometrium (Fig. 7B). To confirm this finding, the uterine samples from the timed-pregnant rat model were also analyzed (Fig. 7C). In Sprague-Dawley rats, the average length of pregnancy is about 23 days. Day16 pregnancy corresponds to the second trimester and day 20-pregnancy corresponds to the third trimester in human pregnancy [7]. On immunoblot, we found that SmAV protein expression increases significantly in the rat at day 16 of pregnancy but continues to rise significantly at day 20. It was noted that SmAV protein levels decreased in the rat in labor, possibly reflecting high level of protease activity and initiation of involution at an accelerated pace in the rat model [29]. Increases in SmAV protein levels in rat myometrium were demonstrated for pregnant day 16, day 20 and in labor samples.

### Myometrial SmAV associates with tyrosine-phosphorylated proteins and ERK in a stretch-dependent manner

**Figure 8:**
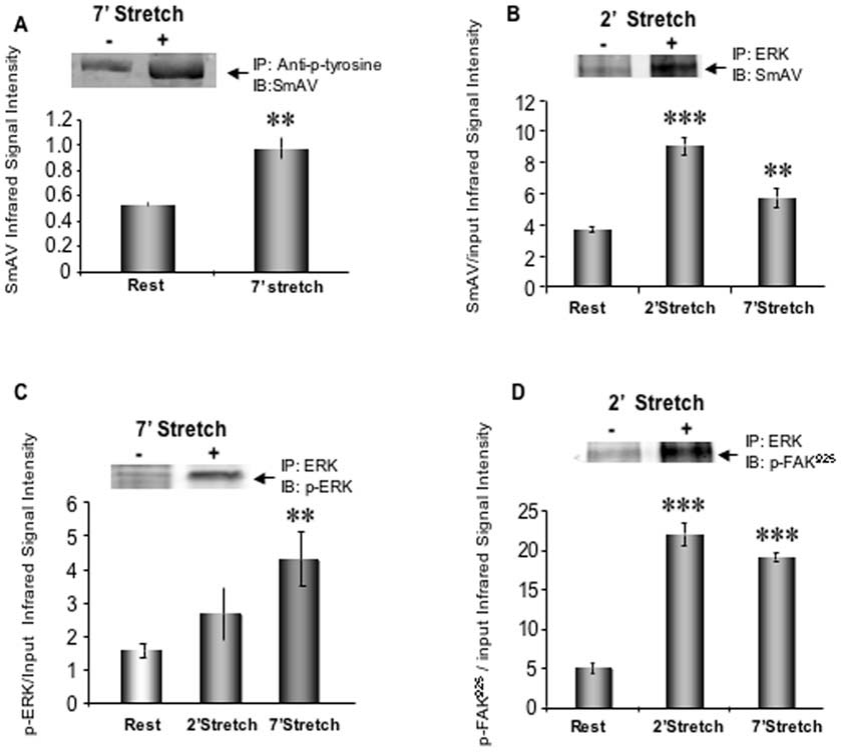
SmAV co-immunoprecipitates with phosphotyrosine, ERK and FAK. A. Myometrial SmAV associates with tyrosine-phosphorylated proteins in a stretch-dependent manner. Stretch and unstretched term pregnant human uterine smooth muscle homogenates were immunoprecipitated (IP) with anti-phospho-tyrosine antibody and immunoblotted (IB) with SmAV antibody. **p<0.01 compared to unstretched control samples. The insert is a typical SmAV western blot with IP samples. n = 5. B. Myometrial SmAV associates with ERK protein in a stretch- dependent manner. Stretched and unstretched term samples were immunoprecipitated with anti- ERK antibody and immunoblotted with anti-SmAV antibody. SmAV densitometry was normalized to input signals. n = 6. **p<0.01, ***p<0.001. C. Confirmation of stretch induced ERK phosphorylation in human myometrium. Stretch and unstretched samples were immunoprecipitated with anti- ERK antibody and immunoblotted with phospho-ERK antibody. The p-ERK2 infrared signals were also normalized to input signals. n = 6. **p<0.01. D. Myometrial ERK associates with FAK in a stretch-dependent manner. Stretch and unstretched samples homogenates were immunoprecipitated with anti- ERK antibody and immunoblotted with phospho-FAK^925^ antibody. The p-FAK infrared signals were normalized to input signals. n = 6. ***p<0.001

Gestational upregulation of SmAV expression implies a possible role of this protein during pregnancy. To explore the possible function of the gestation-induced increases in SmAV expression in the human, we probed anti-phosphotyrosine immunoprecipitates of control and stretched human myometrial strips (term pregnant, stretched 2x slack length for 7 min) for SmAV. Stretch of human myometrial smooth muscle significantly increases the interaction (directly, or indirectly within a macromolecular protein complex) between SmAV and tyrosine phosphorylated proteins (Fig. 8A).

SmAV function has also been linked to ERK activation. There are two ERK binding sites predicted from sequence analysis in the N-terminal end of SmAV [8], [9]. We previously reported that spontaneous labor is associated with phosphorylation and activation of ERK in rat myometrium [7], [10]. In order to test the hypothesis that SmAV acts as a scaffolding protein to recruit ERK in focal adhesion signaling, stretched and unstretched uterine smooth muscle homogenates were immunoprecipitated (IP) with an anti-ERK antibody. Immunoblots were probed with the anti-SmAV antibody. As shown in Fig. 8B and 8C, stretching human pregnant uterine smooth muscle induces a significant increase in ERK-SmAV and the pERK-SmAV interactions. The interactions could be direct or could represent association of both proteins with the adhesion plaque complex. Thus, since phosphorylation of the adhesion plaque protein FAK at residue 925 increases in these samples with stretch, we probed for the association of phospho-FAK-Y925 with ERK and, as is shown in Fig. 8D, this association also increased with stretch.

### Sub-cellular localization of SmAV with the adhesion plaque marker vinculin in pregnant human myometrium

**Figure 9:**
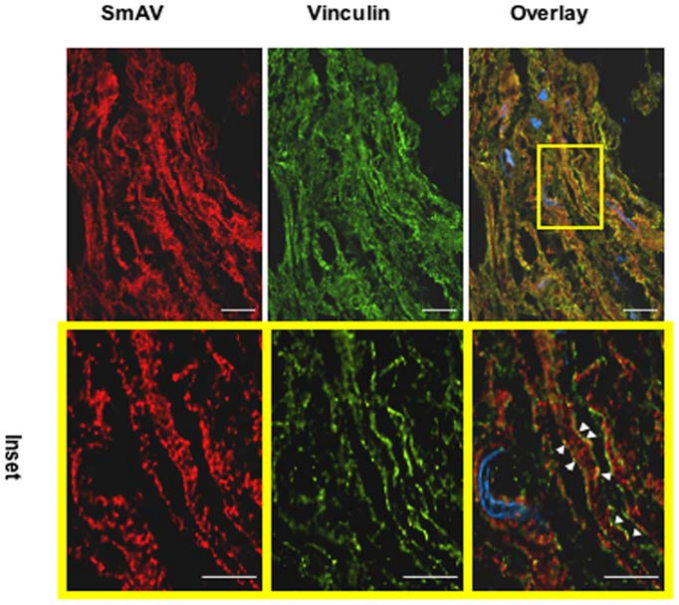
Smooth muscle Archvillin (SmAV) in pregnant human myometrium is localized with vinculin at cell surface. Term, not-in-labor, human uterine smooth muscle strips were microdissected and stretched to 2 fold slack length for 7 minutes. The fresh frozen sections (10 sections for each sample) were stained with rabbit anti-SmAV and mouse anti-vinculin. Alexa-dye-conjugated goat secondary antibodies were used. Left column, SmAV (red); middle column vinculin (green); right column, merged image. Top panels, scale bar, 20 µm. Lower 3 panels, expanded magnification of boxed area in top merged image. SmAV localizes with the dense plaque maker vinculin (inset, white arrows) at cell surface. Scale bar, 10 µm, n = 3.

In vascular smooth muscle cells, SmAV undergoes a stimulus-induced translocation from the core of the cell to the cell cortex [8]. To determine if indeed SmAV colocalizes with adhesion plaques in myometrial cells from pregnant women, we used immunohistochemistry on term, not in labor pregnant myometrial tissue sections to identify the subcellular location of SmAV. As can be seen in Fig. 9, SmAV and vinculin, an adhesion plaque marker, localize at the cell surface after the myometrial strips were stretched for 7 min (inset, arrows).

In summary, as illustrated in Fig. 8, the immunoprecipitation experiments demonstrated interactions between these phosphorylated proteins within focal adhesion complexes and point to a direct mechanistic link between focal adhesions, SmAV and ERK activation.

## Discussion

One of the major findings of the current study is that the role of ERK-mediated phosphorylation of CaD previously predicted from rat studies can be extended to the human. CaD is an actin binding smooth muscle protein that, like troponin in striated muscle, works with tropomyosin to block the binding site of myosin on actin. Upon phosphorylation of the ERK site on CaD, however, a conformational change occurs that increases actin availability for interaction with myosin. We have shown here that not only do CaD protein levels increase with pregnancy, as previously described, [13], [30] but also that CaD phosphorylation increases in myometrial samples from women in labor, compared to those from women not-in-labor. Furthermore, as predicted by the known effects of CaD to inhibit Ca-dependent acto-myosin interactions in vitro, we have directly shown that human myometrial smooth muscle from pregnant women (>37 week, not in labor) exhibits an increased Ca^2+^_i_ threshold for contraction (decreased Ca sensitivity) as CaD levels increase with gestation. In the rat model we have previously shown that an ERK inhibitor delays the onset of RU486-induced preterm labor [7], pointing to a cause-and-effect relationship between ERK and the onset of labor.

Another major finding of the current study is that stretch of human myometrium directly induces CaD phosphorylation in vitro. Although myometrial quiescence in vivo is clinically important for normal pregnancy, it has not been totally clear that how the myometrium switches phenotypically from the “myometrial quiescence” typical of the early pregnant uterus to “myometrial activation” during labor. Similarly, it is known that clinical conditions that increase uterine wall tension stimulate uterine contractions; however, the molecular mechanisms involved have not been clear. Our results support the hypothesis that stretch from the growing fetus at term, when the rate of fetal growth is 7 times that in the first trimester [1], promotes uterine contractions through activation of adhesion plaques and downstream ERK signaling. Furthermore, this stretch-sensitive signaling pathway would help explain the high incidence of preterm labor due to abnormal uterine stretch in conditions such as multiple gestation, polyhydramnios, and macrosomia. Additionally, the increased CaD expression during pregnancy may minimize the level of CaD phosphorylation per mole CaD and counteract the effect of stretch-induced stimuli before the onset of labor.

In the present study we report that expression levels of a newly identified smooth muscle protein, SmAV, increase significantly during pregnancy. SmAV, a known regulator of ERK is likely to facilitate the activation of ERK associated with the onset of labor. Knockdown of SmAV in vascular cells has been shown to inhibit agonist-induced activation of ERK and contraction [8]. We show here that SmAV is biochemically associated with, the adhesion plaque protein FAK in term human myometrium. This association could be direct or, an indirect interaction through binding to other adhesion plaque proteins. Its association with focal adhesion molecules in human myometrium places it in a location where mechanotransduction from the growth of the fetus is sensed and can be transmitted to downstream ERK-dependent pathways including CaD phosphorylation.

In the current study we used an in vitro experimental stretch model to investigate the effect of stretch on ERK, CaD phosphorylation and activation of focal adhesion signaling. Previous studies have shown that myometrial stretch also can activate focal adhesion signaling [3], [4], MAP kinase [31], [32] and regulate expression of transforming growth factor [33]. The current study demonstrates, for the first time the relationship of stretch and CaD phosphorylation in human myometrium. Brief in vitro stretch is clearly different from the in vivo stretch due to fetal growth. This is a limitation of the in vitro stretch model. However, the advantage of in vitro stretch is that it is allows the study of the effect of stretch in the absence of circulating hormones and neuronal inputs. Other investigators also used in vitro stretch models to study the effect of stretching and shortening of human myometrial strips on prostaglandin production. Those data demonstrated a differential prostaglandin synthesis that is related to myometrial stretching and shortening which could also play a role in uterine quiescence during gestation and increased contractility during parturition [34].

**Figure 10:**
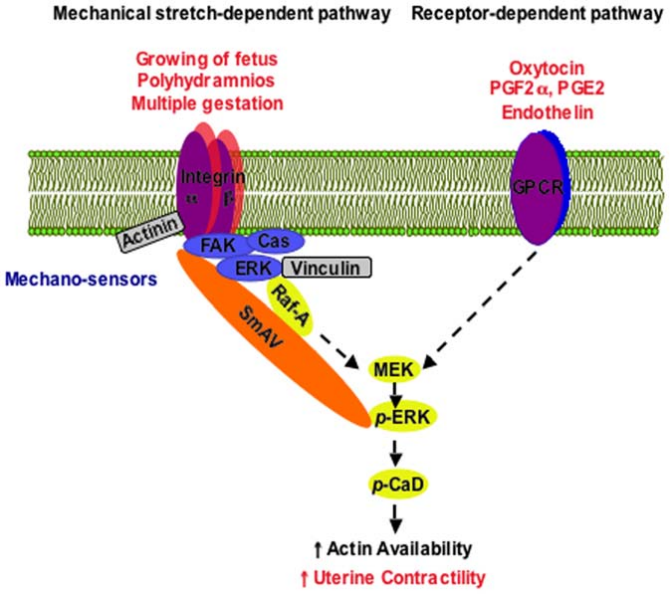
Model of signaling pathways involved in myometrial ERK activation. In addition to classical G-protein coupled receptors (GPCR) pathway described by others, data presented here suggest that the activation of ERK by focal adhesion signaling also promotes myometrial contraction and contributes to the initiation of labor. p: phosphorylation.

Here we have shown that the molecular mechanisms of stretch-induced activation of human myometrium include (1) increased tyrosine phosphorylation of FAK, and Cas, hallmarks of focal adhesion signaling, (2) increased association of, or tyrosine phosphorylation of, α-actinin with adhesion plaque complexes and (3) downstream, stretch-induced increases in ERK and CaD phosphorylation. We have previously shown in the timed pregnant rat model that ERK is targeted to the cell cortex in late term pregnancy [6] and the upregulation of SmAV expression may be one factor that contributes to the targeting of ERK to focal adhesions in a gestation-dependent manner. As illustrated in the model shown in Fig. 10, we suggest that signals mediated through GPCR pathways (such as oxytocin, prostaglandins, endothelin etc) and signals mediated through focal adhesions pathways (growth of fetus, polyhydramnios and multiple gestations) may synergize to activate myometrium and initiate the labor contractions.

Taken together, these results indicate that gestational stretch of human myometrium activates adhesion plaque (dense plaque) signaling, and acting through the ERK scaffold SmAV, recruits ERK to dense plaques, leading to the activation of ERK and the subsequent phosphorylation of CaD to promote myometrial contractility during labor. These results could also identify these signaling molecules as target molecules for drug development aiming in regulation of uterine contractility and interruption of preterm contractions.
